# Reporting and managing ethical issues in intensive care using the critical incident reporting system

**DOI:** 10.1177/09697330241244514

**Published:** 2024-06-07

**Authors:** Tina Hiltunen, Riitta Suhonen, Jaana Inkilä, Helena Leino-Kilpi

**Affiliations:** 8058 University of Turku; 3836 HUS Helsinki University Hospital; 8058 University of Turku; Turku University Hospital, Wellbeing Services County of Southwest Finland; 3836 HUS Helsinki University Hospital; 8058 University of Turku; Turku University Hospital, Wellbeing Services County of Southwest Finland

**Keywords:** Critical incident reporting system, ethical issues, intensive care, nursing

## Abstract

**Background:**

Intensive care nurses frequently encounter ethical issues with potentially severe consequences for nurses, patients, and next of kin. Therefore, ethical issues in intensive care units (ICU) should be recognized and managed.

**Research objectives:**

To analyze ethical issues reported by intensive care nurses and how reported issues were managed within the organization using register data from the HaiPro critical incident reporting system (CIRS), and to explore the suitability of this system for reporting and managing ethical issues.

**Research design:**

This was a retrospective descriptive register study. CIRS reports on ethical issues in adult ICUs (*n* = 12) in one hospital district in Finland over 25 months (2019–2021) were analyzed through inductive content analysis and descriptive quantification. The CIRS’s suitability for reporting and managing ethical issues was evaluated through a strengths, weaknesses, opportunities, and threats (SWOT) analysis.

**Ethical considerations:**

The study was approved by the University Ethics Committee, and permission to conduct the research was granted before data collection within the organization.

**Results:**

CIRS reports on ethical issues (*n* = 35) made by nurses were found in seven of the 12 ICUs. The CIRS managers of these units managed these reports. The ethical issues described by the nurses were divided into four main categories: nature, situational information, consequences, and contributing factors. Management of reported ethical issues was divided into three main categories: preventive actions proposed by nurses, proposals for actions by CIRS managers, and actions taken by CIRS managers.

**Conclusions:**

Systematic register data broadly describe ethical issues and their management, indicating that the CIRS could be suitable for reporting and managing ethical issues, thereby enabling the monitoring and development of ethical quality at the unit and organizational levels.

## Introduction

Intensive care is the care of critically ill patients, which is executed through multi-professional cooperation in a specialized highly technological environment called the intensive care unit (ICU).^1^ Intensive care is ethically challenging due to the decisions about life and death made, the vulnerability of critically ill patients, and the allocation of intensive care resources.^2,3^ Intensive care professionals report experiencing recurring severe ethical issues.^4^ Therefore, the systematic identification, reporting, and management of ethical issues is crucial in ensuring the ethical quality and safety of care.

Healthcare organizations offer different resources to manage ethical issues. In addition to these resources, the reporting and management of ethical issues have been enabled through a critical incident reporting system (CIRS), categorizing ethical issues as critical incidents. Critical incidents are unexpected harmful events posing a threat to patient safety.^5,6^ Through a CIRS, incidents or near misses are reported anonymously and managed systematically to develop patient safety^5,6^ and now also ethical safety. In Finland, a national CIRS called HaiPro was first introduced in 2007^7^ and was complemented in 2017, with the ethical categorization developed by the HUS Helsinki University Hospital in cooperation with Awanic Ltd. Despite the ethical categorization of the HaiPro CIRS becoming increasingly common in Finland, it is still in its early stages, and its use is yet to be reported.

### Background

Ethical issues experienced by intensive care nurses have been described in previous studies vis-à-vis patients, other professionals, or next of kin. Ethical issues related to patients include futile care,^8–11^ unnecessary suffering,^8–10,12^ disrespect of patient autonomy^9,12–14^ or privacy,^10,13,15^ and quality care not enabled regarding access to care,^15^ adequacy of care given,^8^ or the allocation of resources.^9,10,14^ Regarding other professionals, nurses experience ethical issues concerning decision-making,^8,11–13,15^ unprofessional conduct,^14^ and cooperation.^12,15^ Regarding next of kin, ethical issues are experienced during interactions^8,13,14^ or decision-making.^8,11^ The situations where ethical issues occurred were described only briefly in previous studies regarding end-of-life care,^11,15^ organizing of care,^13^ and multi-professional cooperation.^14^ Ethical issues’ consequences have been described more broadly regarding patients,^8,11,13,15^ nurses,^8–13,15,16^ and next of kin.^11^ The most frequently reported consequences were patient suffering^8,11,13,15^ and nurses’ moral distress.^8,10–12,15^

Factors contributing to ethical issues were described in many previous studies regarding insufficient resources^8,11,13^ specified as the lack of resources in general^8^ or regarding nursing personnel, patient beds, or equipment.^13^ Unit culture as a contributing factor was found to relate to the hierarchy of a unit where nurses experienced not being heard when faced with ethical issues.^11^ Unit setting was described as a contributing factor because the care environment is an open space where patient privacy is difficult to maintain.^15^ Preventive actions for ethical issues proposed by nurses included the management of ethical issues^9,11,15,16^ and the development of cooperation^9,11,16^ and ethics education^11,15^ programs promoting the understanding of ethics^11^ and moral development.^15^

The management of ethical issues in hospital settings is implemented as a service offered by the healthcare organization, the unit, or a combination of both. Ethical support services offered by healthcare organizations are clinical ethics consultation services^17,18^ and clinical ethical committees.^19,20^ Through these services, available around the clock, ethical issues are managed by an expert or experts in ethics.^18^ Ethical issues are discussed with those involved, and different perspectives are sought,^17,18,20^ after which recommendations are made for specific cases.^17,18^ However, ethical support services remain underused,^19^ the hierarchy within the system affects the use of services,^20^ and nurses fear the consequences of requesting ethics consultation services.^11^

Group discussions are a form of ethical support organized by units in general (e.g., ethics rounds held twice a month by a nurse ethicist), with the aim of learning from ethically challenging cases.^21^ Ethical support that combines the support organized by a unit and an organization is an ethical decision-making model and an ethical categorization of a CIRS. The ethical decision-making model METAP (Modular, Ethical, Treatment, Allocation, Process) aims to support ethical decision-making by offering different types of ethical support depending on the ethical issue’s severity.^22^ However, the ethical categorization of a CIRS utilizes the reporting system of an organization to report and manage ethical issues at the units. Nurses have described making incident reports as one way to act when faced with ethical issues,^11^ and the ethical categorization of the CIRS has been suggested.^23^

Ethical issues experienced by intensive care nurses have been studied extensively over the last decade, and recurring ethical issues are now known. However, the case-specific descriptive knowledge of ethical issues and their management remains limited. Nurses have a central role in caring and advocating for critically ill patients,^24^ and generally, nurses actively report incidents.^5,6^ Although CIRS are used worldwide and have been studied in various settings,^5,6^ an ethical categorization of such a system is yet to be studied. To address this gap in extant research, CIRS register data were utilized to study ethical issues reported by intensive care nurses and their management. To support further development of CIRS, the suitability of the system for this purpose was evaluated.

### Research objectives and questions

The purpose of this study was twofold: first, to analyze ethical issues reported by intensive care nurses and how reported issues were managed within the organization using register data from the HaiPro CIRS and, second, to explore the suitability of this system for reporting and managing ethical issues.

The research questions are as follows:

1. What ethical issues were reported by intensive care nurses?
2. How were the ethical issues managed within the organization?
3. Is CIRS suitable for reporting and managing ethical issues?

## Methods

### Research design

This was a retrospective descriptive register study^25^ in which ethical issues reported by nurses and the management of reports in adult ICUs over 25 months (2019–2021) were analyzed through inductive content analysis^26^ and descriptive quantification.^27^ Finally, the suitability of the CIRS was evaluated through a strengths, weaknesses, opportunities, and threats (SWOT) analysis^28^ utilizing an organic approach to structure the evaluation.^29^

### HaiPro CIRS

Reports on ethical issues in the HaiPro CIRS correspond to other incidents or near misses. Reports are made anonymously through an electronic form in which the reported incident, the situation in which it happened, and the consequences, as well as the contributing factors and descriptions of how the incident could be prevented, are described in free text. The reporter, who in this study is a nurse, may anonymously leave an email address to enable further inquiries about the incident by the CIRS manager.^30^ The report is managed by CIRS managers who are nurse managers^31^ or physician–nurse pairs responsible for patient safety at the units.^30^ The CIRS managers manage the reports electronically by categorizing the reported issue and by proposing actions and describing actions taken in free text.^31^ Reports classified as serious by CIRS managers are referred to a higher level in the organization and managed multi-professionally.^30^

### Sampling

The study was conducted in adult ICUs using the ethical categorization of the HaiPro CIRS. Purposive sampling was conducted at the hospital district level. From the selected hospital district, all adult ICUs (*n* = 12) were chosen based on the name of the units (intensive, “teho” in Finnish), from a list of all units within the hospital district. Data were collected over 25 months to gain a sample of how the system had been used. Data collection time was evaluated to allow the nurses to familiarize themselves with the ethical categorization in the CIRS and enable the reporting of recurring issues. All reports made by nurses and categorized as ethical by either the nurses or the CIRS managers at the selected units were included.

### Data collection procedure

**Figure 1.**
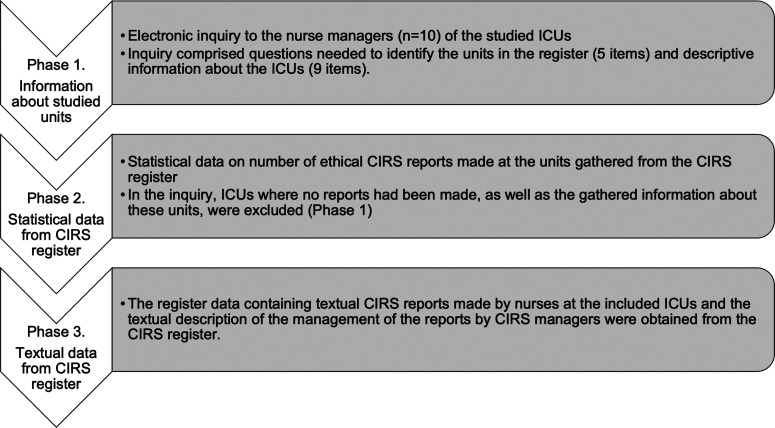
Data collection phases.

Data collection was conducted in three phases, including gathering information about the studied units, statistical data from the CIRS register, and textual data from the CIRS register (Figure 1). After receiving permission from the target hospital district to conduct the research, an inquiry was sent to the nurse managers of the ICUs via email by a contact person within the organization.

The inquiry comprised questions designed to identify the units in the register required to gather register data and descriptive information about the ICUs. Questions to identify the unit included questions regarding changes in the unit during 2019–2021 (one item: no/yes, describe) and unit information (four items: current and previous unit names and identification numbers). Questions regarding descriptive information about the ICUs included numeral data of unit operations (five items: number of beds, patients cared for annually, number of nurses, and nurse-patient ratio during day and night shifts), questions regarding informing or educating nurses about reporting ethical issues through the CIRS (two items: yes/no/cannot tell), and questions regarding ethics as part of the orientation and ethical education offered to nurses in 2019–2021 (two items: yes/no/cannot tell).

### Analysis

The analysis was a multifaceted process comprising the analysis of the descriptive information on the investigated ICUs (A), analysis of textual register data (B), quantification of textual register data (C), and evaluation of the suitability of the CIRS for reporting and managing ethical issues (D). First, descriptive information about the investigated ICUs (A) was presented as means or combined figures to describe the ICUs and their operations. Second, the textual register data were analyzed (B) through inductive content analysis according to Graneheim and Lundman.^26^

Content analysis began by identifying the unit of analysis, which was the written CIRS report, and its management. All reports were assigned an identification number (R1–35) and categorized into five meaning units according to questions answered in different sections of the CIRS, thereby forming three meaning units of text produced by nurses and two meaning units of text produced by the CIRS managers. The data were read several times, after which condensation and coding were performed. Within meaning units, codes were rearranged to form the main categories and subcategories describing the data.

Descriptive quantification of the textual register data (C) was utilized,^27^ generating information about the CIRS register data by counting the number of sections reported by nurses and CIRS managers and by counting the number of times categories were described in the reports. Finally, the suitability of the CIRS for reporting and managing ethical issues was evaluated (D) by the researcher through an organic SWOT analysis as an approach for highlighting insights.^29^ Internal factors (strengths and weaknesses) within the unit and external factors (opportunities and threats) from an organizational perspective were evaluated. The analysis begins by stating the goal, followed by evaluating practice and stating the internal and external factors.^28^

### Ethical considerations

The study followed good research practices and ethical conduct^32^ and was approved by the University Ethics Committee (13/2021). Permission to conduct the research was obtained from the organization (8/2021). The CIRS register data are owned by the organization and can be used for research purposes with permission granted. Ethical aspects especially considered were confidentiality and informing ICU nurses and nurse managers about the research. The CIRS reports were anonymous, and confidentiality was ensured by a contact person from the organization before sending the data to the researcher. To further ensure confidentiality, the researcher did not receive information on when or where the unit-specific reports were made, and the descriptive information about the units was not combined with the register data. Nurses and nurse managers were informed about the study through letters containing information about the study and a guarantee of confidentiality. The information letters were sent electronically to nurse managers in the ICUs, who forwarded them to the nurses.

## Results

The results are presented in four sections: descriptive information, including the description of the investigated ICUs and the CIRS register data; ethical issues reported; management of ethical issues; and the suitability of a CIRS for reporting and managing ethical issues.

### Descriptive information

#### Description of the investigated ICUs

Ethical issues were reported in seven units (7/12, 58 %), including the units in this study. Nurse managers from all the included units answered the inquiry. The ICUs had 82 beds. Approximately 9900 patients are cared for annually in these units. A total of 525 nurses worked in the ICUs, and the reported nurse-patient ratio during the day and night shifts was 1:1 in five units and 1:2 in two units.

These units are responsible for informing and instructing nurses to report ethical issues through the CIRS. Five units (71%) reported that they had informed nurses of the possibility of reporting ethical issues, and in four units (57%), the nurses had been instructed on how to make reports. In four units (57%), ethics was part of the orientation for new nurses working in the units, and in three units (43%), ethics education was offered to nursing staff in 2019–2021.

#### Description of the CIRS register data

During the 25-month study period, 35 CIRS reports on ethical issues were created by nurses. These included three sections: description of the event, contributing factors, and prevention of the event. The number of reported sections varied (Table 1).

**Table 1.** Sections of CIRS reports described by nurses and CIRS managers.

Reporter and section of the report	Described in reports, *n* (%)
Nurse	
Description of the event	35 (100)
Contributing factors	19 (54.3)
Prevention of the event	25 (71.4)
CIRS manager	
Proposal for actions	21 (60)
Actions taken	21 (60)

The management of the reports was described by CIRS managers in two separate sections: proposals for actions and actions taken, which were reported in 60 % (*n* = 21) of the reports (Table 1). However, in 83 % (*n* = 29) of the reports, at least one management section contained a written comment from the CIRS manager (Table 2). The length of the reports varied; the mean length of the text in the CIRS report was 119.3 words written by the nurse and 33.7 words written by the CIRS manager.

**Table 2.** Sections of management described by CIRS managers.

Section of management	Described in reports, *n* (%)
Both proposals for actions and actions taken	13 (37.1)
Only proposal for actions	8 (22.9)
Only actions taken	8 (22.9)
No management	6 (17.1)

### Ethical issues reported

Ethical issues reported by the nurses were divided into four main categories: nature, situational information, consequences, and contributing factors (Table 3).

**Table 3.** Ethical issues reported by nurses through the CIRS.

Main category	Category	n*	Subcategory
Nature of ethical issues	Regarding patients	29	Patient rights not fulfilled
			Unnecessary suffering
	Regarding professionals	13	Neglected obligations
			Disrespectable behavior
	Regarding next of kin	5	Communication issues
			Decision-making issues
Situational information on ethical issues	Related to patient care	20	Nursing
			Visitation
			Scientific research
	Related to unit operations	13	Conduct of colleague
			Execution of data protection
			Insufficient resources
	Related to multi-professional cooperation	12	Participation in decision-making
			Communication
Consequences of the ethical issues	Consequences to the patient	33	Harm caused to the patient
			Violation of patient’s rights
	Consequences to the nurse	18	Nurse unable to act according to guidelines
			Effect on nurses’ well-being at work
	Consequences to the next of kin	2	Confusion about the situation
			Right to privacy was violated
Contributing factors to the ethical issues	Factors related to the event	17	Unit
			Nurse
			Physician
			Patient
	Factors related to event conditions	12	Situation in the unit
			Time of day

n* = number of times the category was described in the reports.

Nurses described the nature of the ethical issues (*n* = 47) in the description of the event, including ethical issues regarding patients, professionals, and next of kin. Two or more ethical issues were described in ten reports. Patient-related ethical issues were most frequently reported, including patients’ rights to autonomy, privacy, the right to good care not being fulfilled, and unnecessary suffering, described as causing unnecessary pain or prolonging suffering. As reported by a nurse:

“…the patient had low blood pressure and had a Noradrenalin infusion that was not stopped even though it was already clear that the patient was going to die. The patient's suffering was unnecessarily prolonged” (R23)

Ethical issues regarding professionals were described as neglected obligations regarding nurses not making incident reports or physicians not responding to nurses’ calls for help. Disrespectful behavior was defined as professionals acting disrespectfully towards patients or nurses. Next-of-kin-related ethical issues were regarding communication (e.g., not providing truthful information or providing information in an unsuitable situation) and decision-making when the patient’s will conflicted with the requests of their next of kin or when their participation in decision-making negatively affected patient care. As described by a nurse:

“Care was continued over the weekend due to a difficult family situation.” (R2)

Situational information on ethical issues (*n* = 45) was included in the description of the event, describing situations in which ethical issues occurred regarding patient care, unit operations, and multi-professional cooperation. Patient care-related situations regarding nursing included the care of the critically ill, dying, awake, or delirious patients. Ethical issues also occurred when the patient’s care was prolonged or when the patient underwent an operation or procedure. Patient care-related situations regarding visitations or scientific research conducted in the unit were also described. As reported by a nurse:

“…research conducted on an old man, who does not even know why he is in hospital or what is the purpose of his care.” (R19).

Unit operations-related situations described included the conduct of colleagues (e.g., disrespectful behavior or the violation of privacy), the execution of data protection in the handling of data protection waste, and insufficient nursing resources to meet patients’ needs. Multi-professional cooperation-related situations were reported regarding decision-making, including uncertainty about the care plan and aspects related to communication between nurses and physicians regarding prescriptions. As described by a nurse:

”The anesthesiologist was informed so that required changes to the drug administration could have been made…The anesthesiologist blurted that the nurse should make the changes…” (R32)

The consequences of ethical issues (*n* = 53) were reported as part of the description of the event regarding patients, nurses, and next of kin. Consequences to patients were reported most frequently, with the harm caused to the patient described as unnecessary suffering, harm caused by scientific research, unnecessary risks, delayed treatment, and insufficient support. Consequences to patients were also described as a violation of patient rights to privacy, autonomy, and respectful treatment. As reported by a nurse:

“It remained unclear to the nurses if the patient's will was heard regarding the operation?” (R26)

The consequences to nurses were described as feeling unable to act according to the guidelines because of a lack of resources, care instructions, or necessary cannulas or catheters. The nurses reported that their well-being was affected by participating in situations in which their safety was not considered or by acting against their values. As reported by a nurse:

“As nurses, we find this kind of ‘forcible care’ and prolonging very hard because death should be dignified… as nurses, we have to watch these patients all the time, and we see the prolonging and the suffering.” (R6)

Consequences to the next of kin included confusion about the patient’s care when decisions had been made about terminating care, but this was not acted upon as agreed. The next of kin’s right to privacy was violated when information about the patient was provided in front of outsiders.

The factors contributing to ethical issues (*n* = 29) were related to the event and its conditions. Factors related to events regarding units, nurses, physicians, and patients were reported. Unit-related factors included difficulties in managing visits and regarding the organization of care, reported as uncertainty about the patient’s treatment options when care is withdrawn or deviates from the protocol. Nurse-related factors included haste in caring for the patient, fear of the consequences of reporting incidents, and difficulties in evaluating a delirious patient’s condition. Physician-related factors were that the physician was tired, impatient, delayed seeing the patient, provided insufficient care instructions, and did not notice the patient’s pain. Patient-related factors included prolonged care, language barriers, and difficult family relationships. As reported by a nurse:

“Normally the intensive care patient gets a tracheostomy on the 7^th^ day of care if care is prolonged! Now it was delayed till the 13^th^ day of care.” (R1)

Factors related to event conditions were identified as the situation in the unit and time of day. The situation in the unit was reported to be calm or abnormal due to the COVID-19 pandemic or the implementation of new technology. The time of day was reported to be a contributing factor during night shifts, shift changes, and when the physician was on call. As described in a report:

“Happened during the nightshift, physician tired?” (R4)

### Management of ethical issues

The management of ethical issues included data provided by nurses (proposed preventive actions) and CIRS managers (proposals for actions and actions taken) (Table 4).

**Table 4.** Management of the ethical issues.

Main category	Category	n*	Subcategory
Preventive actions proposed by nurses	Development of operations	14	Operational models
			Cooperation
	Reminding of obligations	9	Obligations of all professionals
			Physicians’ obligations
	Enabling quality nursing care	8	In patient care
			Regarding scientific research
Proposal for actions by CIRS managers	Discussion about the incident	15	In the unit
			With other professionals
			Whit the ones involved
	Affecting operations models	9	Reminding of operations model
			Development of operations model
	Informing about the incident	8	To the unit
			To other professionals
Actions taken described by CIRS managers	Discussion about the incident	19	In the unit
			With other professionals
			Whit the ones involved
	Informing about the incident	10	To the unit
			To other professionals
	Affecting operations models	8	Reminding of operations model
			Development of operations model

n* = number of times the category was described in the reports.

Nurses proposed preventive actions (*n* = 31), including the development of operations, reminding of obligations, and enabling quality nursing care. The development of operations involved operations models for gathering information about the next of kin, data protection, end-of-life care, and paying attention to patients. Nurses proposed discussions in the units and agreements on the order and division of tasks. As described by a nurse:

“Cohesive care guidelines, because every patient deserves good palliative care.” (R34)

Regarding all professionals, the reminding of obligations concerning confidentiality, reporting critical incidents, and supporting patient autonomy was proposed. Regarding physicians’ obligations, nurses reported that physicians were obligated to make decisions regarding patient care and that they should be available. As described by a nurse:

“The anesthesiologist should make clear prescriptions and be at hand.” (R16)

Enabling quality care was proposed in patient care and scientific research. Regarding patient care, the nurses proposed precise documentation, precision in making prescriptions, and calm ways of providing care. Nurses also mentioned enabling patient care according to the protocol and with sufficient resources. Regarding scientific research, nurses proposed the assessment of harm caused to patients as a result of scientific research.

CIRS managers made proposals for actions (*n* = 32) and described actions taken (*n* = 37), including discussions about the incident, informing about the incident, and affecting operations models. No specific management strategies were applied to certain ethical issues. Discussions about the incident were proposed and held at the unit’s ward meeting, as a general discussion at the ward, or as a multi-professional discussion about ethical issues. A description of who initiated the discussion or how the issue was discussed was not provided. The managers held discussions with other professionals. Discussions with those involved were also held by the manager of the unit, aiming to hear the parties involved or intervene in the situation. As described by a CIRS manager:

“The matter was discussed with those involved.” (R29)

Professionals working in the unit were informed about the incident through a weekly newsletter. Other professionals were also informed about the incident in accordance with the organization’s policy. Affecting operations models were described as reminding of operations models in terms of documentation, prescriptions, professional confidentiality, consideration of the patient’s pain, care of a patient with a do-not-resuscitate (DNR) order, and end-of-life care. Reminding of operations models was also described regarding how to act if worried about patients participating in scientific research. Operations models were affected through the development of operations models regarding respecting patient autonomy, privacy policies in the unit, end-of-life care, multi-professional cooperation, and an emphasis on professional confidentiality during the orientation of new nurses. As described by a CIRS manager:

“…how can we in the future, for example, by developing intensive care operations models, ensure that the patient’s will comes to the knowledge of all included in the patient’s care and is taken into account during care decisions?” (R26)

### Suitability of a CIRS for reporting and managing ethical issues

**Figure 2.**
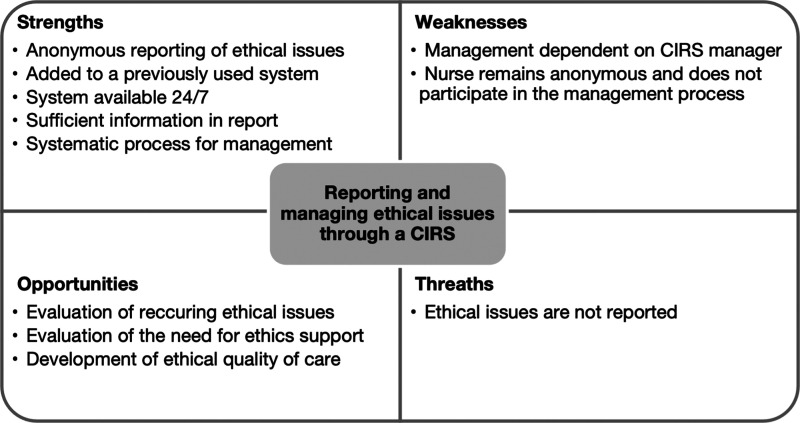
SWOT analysis of a CIRS for reporting and managing ethical issues.

This SWOT analysis aimed to evaluate the suitability of a CIRS for reporting and managing ethical issues based on its strengths, weaknesses, opportunities, and threats (Figure 2). The strength of the CIRS is that it enables anonymous reporting of ethical issues and management within the unit through a system that is available 24/7, has previously been used, and is known to personnel. The information described in this report is sufficient to identify and manage ethical issues. However, a weakness of the system is that the management of reported issues is mostly dependent on CIRS managers who are not necessarily used to managing ethical issues. Additionally, nurses maintain anonymity when reporting ethical issues, and therefore, they do not participate in the management process. However, they propose preventive measures and may anonymously leave an email address to enable contact by the CIRS manager for additional information. At the organizational level, opportunities regarding the CIRS are that the reports remain systematic register data. However, if ethical issues are not reported, the data will not be generated.

## Discussion

This study provides novel findings on the ethical issues reported by intensive care nurses through a CIRS and how these issues were managed. The ethical issues reported mostly corresponded with issues described in previous research regarding patients,^8–15^ professionals^8,11–15^ and next of kin.^8,11,13,14^ However, ethical issues describing professionals neglecting their obligations were not directly mentioned in previous studies. In addition to the situational information described previously,^11,13–15^ unit operations-related situations were reported, and in general, this study provided a more detailed description of situations where ethical issues had occurred.

Consequences were described most in reports. The CIRS prompts the description of consequences,^30^ but it could also indicate that the severity of issues may be easily described through their consequences. The consequences described corresponded with those presented in previous studies^8–13,15,16^ although nurses’ moral distress^8,10–12,15^ was not mentioned directly in this study. However, nurses’ descriptions of how acting against their values affected their well-being at work indicated experiences of moral distress. Contributing factors to ethical issues correlated with the unit-related factors mentioned in previous studies to some extent,^8,11,13,15^ and they generated additional insight into the contributing factors related to nurses, physicians, and patients, as well as event conditions, such as the time of day.

To manage these issues, nurses proposed preventive actions, including the development of cooperation, as mentioned in previous studies.^9,11,16^ One preventive action described in previous studies includes the management of ethical issues,^9,11,15,16^ and reporting itself can be perceived as a preventive measure because it enables the management of, and possibly, learning from the ethical issues experienced. The management of ethical issues was described by the CIRS manager, and the most common way to manage reported ethical issues was through discussions about the incident, similar to other forms of ethics support.^17,18,20–22^ Additionally, informing about the incident brought ethical issues to the attention of ICU professionals. There were no clear patterns as to what management strategies were used for certain ethical issues. Additionally, descriptions of management were brief and remained general.

The suitability of the CIRS for reporting and managing ethical issues has not been previously discussed. The CIRS has been suggested as a way to improve the ethical quality of care.^23^ CIRS managers had made a written comment regarding at least one section in 83 % (*n* = 29) of the reports, which is significantly higher than that in previous studies on all HaiPro CIRS reports.^31^ This may indicate that the management of ethical issues is perceived as important or that incident reports in general are managed actively in ICUs. Generally, severe incident reports are referred to higher up in the organization.^30^ The ethical categorization of a CIRS has been suggested to be combined with other ethics support services.^23^ However, there was no description of the use of additional ethics support services in this study. This could be because only a few healthcare organizations in Finland offer ethics support services,^33^ or because offered services were not utilized or had not been documented.

Nurses have described fearing the consequences of requesting ethics consultations,^11^ and anonymous reporting through the CIRS may ease the reporting of ethical issues. Ethical issues reported mostly corresponded with those described in previous studies, which may indicate that nurses react to ethical issues by reporting them through the CIRS, as described previously.^11^ However, generally the underreporting of incidents is an issue.^5^

This study has implications for future research on the ethical categorization of the CIRS and the use of ethical CIRS register data. Further research is required to determine how nurses and CIRS managers experience the use of this system. Barriers to and facilitators of reporting ethical issues and how ethical issues are managed should be studied. Knowledge is required regarding the competence of CIRS managers in managing reported ethical issues, dividing tasks throughout the management process, and using other ethics support services. The second implication concerns the use of the CIRS register data to evaluate recurring ethical issues in intensive care and possible correlational factors. Register data could also enable further research on reporting practices and more systematic and justified management of reported ethical issues. The possibility of using such data to evaluate ethical quality and the fulfillment of patients’ rights should be investigated.

## Limitations

This study has some methodological limitations. The use of register data poses some limitations because detailed data may be difficult to obtain.^34^ The CIRS register data provided detailed descriptions of the ethical issues reported, but many of the reports had missing sections. Only textual CIRS data was utilized, excluding the categorizations of the report by the CIRS managers.^30^ Additionally, information on CIRS managers or ethics support services offered at the hospital district was not gathered. SWOT analysis has been used in healthcare research previously.^29^ However, in this study, the use of SWOT analysis was limited to a non-regulated organic approach and external evaluation, thereby providing only a general evaluation of suitability. The reported ethical issues mostly corresponded with those described in previous studies, and therefore, they can be used to describe ethical issues reported through a CIRS in adult ICUs in Finland and internationally. However, in accordance with the purpose of the study, the results cannot be generalized.

## Conclusions

This study confirmed that nurses experience various ethical issues in intensive care and that the CIRS could be a suitable, systematic method for reporting and managing ethical issues. CIRS data describing ethical issues can be used to identify issues, evaluate practices, and lead development measures on ethical quality in healthcare from unit to organizational levels.
